# Complete Genome Sequence of *Bacillus subtilis* Phage φ105

**DOI:** 10.1128/genomeA.00641-13

**Published:** 2013-09-05

**Authors:** Daniel R. Zeigler

**Affiliations:** Department of Microbiology, The Ohio State University, Columbus, Ohio, USA

## Abstract

A complete 39,318-bp genome sequence containing 52 coding sequences has been determined for the *Bacillus subtilis* temperate phage φ105. In a lysogen, *B. subtilis* strain 1L32, the φ105 prophage interrupts the *radC* locus, a part of the competence-induced ComK regulon.

## GENOME ANNOUNCEMENT

Bacteriophage φ105, a well-studied temperate phage of *Bacillus subtilis* (1, 2), is capable of specialized transduction and has been developed into a system for cloning and expressing genes in this host (3, 4). Here I describe the complete genome resequencing of φ105.

The approximate chromosomal integration site for φ105 has been previously determined by hybridization studies (5). Genomic DNA from a *B. subtilis* φ105 lysogen, BGSC strain number 1L32, was sequenced in its prophage region by a primer-walking strategy. A total of 435 overlapping PCR fragments were sequenced at the Plant-Microbe Genomics Facility at the Ohio State University on an Applied Biosystems 3730 DNA analyzer using BigDye terminator cycle sequencing chemistry. Average coverage was 9.3×.

The φ105 genome is a linear 39,318-bp double-stranded DNA molecule capable of circularizing through 7-bp cohesive ends. There are 52 predicted coding sequences (CDS). The present sequence differs in 68 nucleotides (nt) from an unpublished genome sequence in the database (AB016282), correcting 15 probable frameshift errors in the older sequence and locating the phage attachment site for the first time. The φ105 prophage interrupts the *radC* gene in the *B. subtilis* 1L32 genome at a 10-bp attachment site (TGTTTATACTT). The *radC* gene is known to be transcriptionally activated by the master regulator of competence development in *B. subtilis*, ComK (6, 7). This observation may explain the so-called “naïve” phenotype of φ105: the prophage is induced not only by DNA-damaging agents, but also by competence development (8).

### Nucleotide sequence accession numbers.

The complete genome sequence is deposited in GenBank under the accession no. HM072038. The version described in this paper is the first version, HM072038.1.
